# Pickled Vegetable and Salted Fish Intake and the Risk of Gastric Cancer: Two Prospective Cohort Studies and a Meta-Analysis

**DOI:** 10.3390/cancers12040996

**Published:** 2020-04-17

**Authors:** Jin Young Yoo, Hyun Jeong Cho, Sungji Moon, Jeoungbin Choi, Sangjun Lee, Choonghyun Ahn, Keun-Young Yoo, Inah Kim, Kwang-Pil Ko, Jung Eun Lee, Sue K. Park

**Affiliations:** 1 Department of Food and Nutrition, Seoul National University, Seoul 08826, Korea; yjy2601@snu.ac.kr (J.Y.Y.); 92hyunjung@snu.ac.kr (H.J.C.); 2 Department of Preventive Medicine, Seoul National University College of Medicine, Seoul 03080, Korea; kajaman3@snu.ac.kr (S.M.); jbchoi@snu.ac.kr (J.C.); sjunlee@snu.ac.kr (S.L.); agkdc@snu.ac.kr (C.A.); kyyoo@snu.ac.kr (K.-Y.Y.); suepark@snu.ac.kr (S.K.P.); 3 Cancer Research Institute, Seoul National University, Seoul 03080, Korea; 4 Interdisciplinary Program in Cancer Biology Major, Seoul National University College of Medicine, Seoul 03080, Korea; 5 Department of Biomedical Science, Seoul National University Graduate School, Seoul 03080, Korea; 6 Department of Occupational and Environmental Medicine, Hanyang University College of Medicine, Seoul 04763, Korea; inahkim@hanyang.ac.kr; 7 Department of Preventive Medicine, Gachon University College of Medicine, Incheon 21565, Korea; kpko@gachon.ac.kr; 8 Research Institute of Human Ecology, Seoul National University, Seoul 08826, Korea

**Keywords:** pickled vegetable, salted fish, gastric cancer, meta-analysis

## Abstract

An increased risk of gastric cancer for pickled vegetable and salted fish intake has been suggested, yet the lack of a dose-response association warrants a quantitative analysis. We conducted a meta-analysis, combining results from our analysis of two large Korean cohort studies and those from previous prospective cohort studies. We investigated the association of pickled vegetable and salted fish intake with gastric cancer in the Korean Genome Epidemiology Study and the Korean Multi-center Cancer Cohort Study using Cox proportional hazard models. We then searched for observational studies published until November 2019 and conducted both dose-response and categorical meta-analyses. The pooled relative risk (RR) of gastric cancer incidence was 1.15 (95% Confidence Interval (CI), 1.07–1.23) for 40 g/day increment in pickled vegetable intake in a dose-response manner (*P* for nonlinearity = 0.11). As for salted fish intake, the pooled risk of gastric cancer incidence was 1.17 (95% CI, 0.99–1.38) times higher, comparing the highest to the lowest intake. Our findings supported the evidence that high intake of pickled vegetable and salted fish is associated with elevated risk of gastric cancer incidence.

## 1. Introduction

Gastric cancer, the fifth most common cancer, is the third most common cause of death from cancer. Among Eastern Asian countries, where gastric cancer ranks the second highest for both incidence and cancer mortality, South Korea has the highest rate of gastric cancer in both sexes (age-standardized incidence rate of 39.6 and mortality rate of 7.0 per 100,000 population in 2018) [1,2,3]. Despite advanced treatments targeting gastric cancer, it remains a life-threatening neoplasm worldwide [4]. Gastric cancer is a multifactorial disease, with several environmental factors such as diet and *Helicobacter pylori* (*H. pylori*) infection contributing to the carcinogenesis [5]. The synergistic effect between dietary salt intake and *H. pylori* infection, a class I carcinogen of gastric cancer, has been previously observed [6,7,8]. Dietary salt intake induces mucosal damage and gastric cell proliferation, enabling the subsequent *H. pylori* colonization and infection [9]. *H. pylori* infection causes gastric inflammation and epithelial damage, eventually increasing the risk of precancerous lesions or gastric cancer [10]. The World Cancer Research Fund (WCRF) proposed “foods preserved by salting” as probable risk factors of gastric cancer, as evidenced from epidemiological studies, referring mainly to pickled vegetables and salted fish [11]. Although previous meta-analyses observed an increased risk of gastric cancer for high intake of pickled vegetables or salted fish, evidence on a dose-response association remains equivocal [12,13]. The average daily intake of *kimchi*, a traditional Korean side dish of salted and fermented vegetable, in Korea (127.72 g/day) far exceeds that of *tsukemono*, a typical Japanese pickle, in Japan (10.96 g/day), warranting the need for studies on pickled vegetable intake among Korean population [14,15]. A recent meta-analysis of Korean case-control studies suggested an increased gastric cancer risk for high intake of *kimchi*, but the results are subject to recall bias or selection bias [16]. Despite gastric cancer ranking the highest in both incidence and cancer mortality in Korea, the majority of the cohort studies included in previous meta-analyses were based on Japanese or Chinese population. Further Korean prospective cohort studies are warranted to establish a conclusive association of the intake of pickled vegetables and salted fish with gastric cancer risk.

Therefore, we investigated the association of pickled vegetable and salted fish intake with the risk of gastric cancer incidence and mortality in two large Korean cohort studies, the Korean Genome Epidemiology Study (KoGES) and the Korean Multi-center Cancer Cohort (KMCC). We then conducted a meta-analysis to combine our results with the risk estimates extracted from prospective cohort studies. We hypothesized that high intake of pickled vegetables and salted fish is associated with an increased risk of gastric cancer.

## 2. Materials and Methods

### 2.1. Longitudinal Analysis of Two Korean Cohort Studies

#### 2.1.1. Study Population

The KoGES, a consortium project of six prospective cohort studies, consists of three population-based cohorts, the Ansan and Ansung study, the Cardiovascular Disease Association Study (CAVAS), and the Health Examinee (HEXA) study. The three population-based cohorts consist of community-dwellers and participants, aged 40 years or older at baseline, who were recruited from the national health examinee registry. The participants were recruited between 2001 and 2002 for the Ansan and Ansung study, 2005 and 2011 for the CAVAS, and 2004 and 2013 for the HEXA study. A detailed explanation of the study design is reported elsewhere [17]. We excluded participants who did not answer any items on the food frequency questionnaire (FFQ) (*n* = 1339); those who did not answer more than 11 items on the FFQ (*n* = 1603); those who did not answer all items related to rice on the FFQ (*n* = 668); those who did not answer pickled vegetable or salted fish items on the FFQ (*n* = 1375 for pickled vegetables; *n* = 572 for salted fish); those who did not provide consent for the linkage to the vital registration data (*n* = 1291 for pickled vegetables; *n* = 1299 for salted fish); those who were previously diagnosed with cancer (*n* = 6089 for pickled vegetables; *n* = 6107 for salted fish); those with missing or implausible level of energy intake (> 3 standard deviations from the natural logarithm transformed mean) (*n* = 2355 for pickled vegetables; *n* = 2365 for salted fish); and those who died within three years from the baseline (*n* = 1367 for pickled vegetables; *n* = 1374 for salted fish). The number of eligible participants differed between the analysis of pickled vegetable intake and that of salted fish intake due to a discrepancy in the number of participants who did not answer corresponding items on the FFQ. After exclusion, a total of 195,624 participants were included in the analysis of pickled vegetable intake with gastric cancer mortality, and 196,384 participants were included in the analysis of salted fish intake with gastric cancer mortality. The study protocol was approved by the Institutional Review Board of the Korea Centers for Disease Control and Prevention (gcirb2017-381). Written informed consent was obtained from all study participants before the data collection.

The KMCC, a multi-center prospective cohort study, consists of cancer screening volunteers, who were recruited from four urban and rural areas (Haman, Chungju, Uljin, and Pohang) between 1993 and 2004. Further details on the study design have been presented elsewhere [18]. For the analysis of gastric cancer incidence, we excluded participants who did not answer pickled vegetable or salted fish items (*n* = 16,005 for pickled vegetables; *n* = 9066 for salted fish); one participant under 20 years old; and those who were previously diagnosed with cancer (*n* = 112 for pickled vegetables; *n* = 242 for salted fish). For the analysis of gastric cancer mortality, we further excluded participants who died within three years from the baseline (*n* = 321). After screening, a total of 4513 participants were eligible for examining the association of pickled vegetable intake with gastric cancer incidence, and 11,322 participants were eligible for examining the association of salted fish intake with gastric cancer incidence. As for the analysis of the association of gastric cancer mortality with salted fish intake, a total of 11,001 participants were included. There were not enough gastric cancer deaths to conduct the analysis with pickled vegetable intake. The study protocol was approved by the Institutional Review Boards of Seoul National University Hospital (H-01-10-084-002 and H-1310-082-528) and performed in accordance with the principles of the Declaration of Helsinki. Written informed consent was obtained from all study participants.

#### 2.1.2. Assessments of Diet and Covariates

Diet assessment was conducted through a 103-item semi-quantitative FFQ in the Ansan and Ansung study and through a 106-item semi-quantitative FFQ in the CAVAS and the HEXA study. Detailed information on the FFQ validation study is stated elsewhere [19,20]. We considered *kimchi*, and *jangajji* (non-fermented pickled vegetables) as pickled vegetables and salted mackerel and *jeotgal* (salted seafood) as salted fish. The FFQ asked intake frequencies in nine categories (almost never, once/month, 2–3 times/month, 1–2 times/week, 3–4 times/week, 5–6 times/week, once/day, twice/day, and 3 times/day) and portion sizes in three categories (less than standard, standard, and more than standard). We calculated the intake in g/day by multiplying the frequency by the standard portion size. Trained interviewers additionally inquired on smoking status (never, past, and current smoker), the number of cigarettes smoked daily, and the number of smoking years. We calculated pack-years by dividing the daily number of cigarettes by 20 and multiplying this by the number of years smoked. The participants also provided their alcohol drinking status (never, past, and current drinker), frequency, and amount in glasses for various types of alcohol. We estimated the total ethanol intake by summing up the product of the frequency and amount and applying the standard ethanol content per glass [21,22]. Body mass index (BMI) was calculated as the weight in kilograms divided by the square of the height in meters.

Dietary habits and lifestyle factors were assessed through a structured questionnaire in the KMCC. The questionnaire asked about the intake frequencies in four categories (never, 3–4 times/month, 3–4 times/week, and almost daily) between 1993 and 2001 and in eight categories (never, once/month, 2–3 times/month, once/week, 2–3 times/week, 4–6 times/week, daily, and more than 2 times/day) between 2002 and 2004. We categorized salt-preserved vegetables, *kimchi*, and *mu-kimchi* (radish *kimchi*) as pickled vegetables and salt-preserved fish as salted fish. Because the questionnaire did not examine the portion size, we calculated the intake in g/day by multiplying the frequency by one serving unit, which was defined as 40 g/day for pickled vegetables as published in the WCRF Third Expert Report and 60 g/day for salted fish based on the Korean Dietary Reference Intake [11,23]. The participants additionally provided their smoking status (never, past, and current smoker) and the alcohol drinking frequency. BMI was calculated using the weight and height measured during the physical examination.

#### 2.1.3. Statistical Analysis of the KoGES and the KMCC

In the analysis of gastric cancer mortality, person-time was calculated from the baseline date to gastric cancer death, or the end of the follow-up (December 31, 2015 for the KoGES and December 31, 2014 for the KMCC), whichever came first. For the analysis of gastric cancer incidence in the KMCC, we calculated the person-time from the baseline to the date of the gastric cancer diagnosis, death, or the end of the follow-up (December 31, 2014), whichever came first. We conducted the analysis for the KoGES and the KMCC separately using Cox proportional hazard models and pooled the results using a fixed effect model [24,25].

We tested the assumption for proportionality of hazard using interaction terms between the person-time and intake of pickled vegetables or salted fish and observed no violation. The regression model for the KoGES was stratified by age at baseline (40 to <50, 50 to <60, and 60+ years) and the study cohort (the Ansan and Ansung study, the CAVAS, and the HEXA study) and further adjusted for age at baseline (continuous, year), sex, total energy intake (continuous, kcal/day), survey year (continuous, year), BMI (10 to < 18.5, 18.5 to < 23, 23 to < 25, 25 to < 30, and 30+ kg/m^2^), smoking status (0, 0 < to < 10, 10 to < 20, and 20+ pack-years), and alcohol intake (0, 0 to <5, 5 to <15, 15 to <30, and 30+ g/day). The KMCC model was stratified by age at baseline (20 to <50, 50 to <60, 60 to <70, and 70+ years) and further adjusted for age at baseline (continuous, years), sex, survey year (continuous, year), BMI (10 to <18.5, 18.5 to <23, 23 to <25, 25 to <30, and 30+ kg/m^2^ for the analysis of gastric cancer incidence and 10 to <23, 23 to <25, and 25+ kg/m^2^ for the analysis of gastric cancer mortality), smoking status (never, past, and current smoker), and the alcohol drinking frequency (never, 0< to <1 time/week, 1 time/week to <3 times/week, 3 times/week to <1 time/day, and 1+ time/day). For the analysis of pickled vegetable intake, we conducted a sensitivity analysis by further adjusting the intake of fresh vegetables (g/day in tertile in the KoGES and <1 time/month, 1 time/month to <1 time/day, and 1+ time/day in the KMCC). We estimated the relative risks (RRs) and 95% confidence intervals (CIs) according to the quantile of pickled vegetable and salted fish intake as well as per one serving increment. A test for linear trend was conducted by treating the median value of each quantile as a continuous variable in the model. We further estimated the sex-specific RRs and included the results in the subgroup meta-analysis. All statistical analyses were performed using SAS software, version 9.4 (SAS Institute Inc, Cary, North Carolina) based on a significance level of 0.05.

### 2.2. Meta-Analysis of the Cohort Studies

#### 2.2.1. Data Sources

The overall process of the meta-analysis was performed and reported according to the Meta-analyses Of Observational Studies in Epidemiology (MOOSE) guideline [26]. We searched for observational studies published until November 2019 from PubMed, EMBASE, and KoreaMed using search terms relevant to ‘diet’, ‘pickled vegetable’, ‘salted fish’, ‘gastric cancer incidence’, and ‘gastric cancer mortality’ (Table S1). We additionally retrieved studies by manually searching the reference lists of relevant studies or recent meta-analyses.

#### 2.2.2. Study Selection

Two researchers (JY Yoo, HJ Cho) initially screened the studies by title and abstract and conducted a full-text review of possibly eligible studies. A third person (JE Lee) checked the screening process, and any uncertainties were discussed and resolved at a weekly meeting. The eligibility criteria for the study selection varied depending on whether the studies were included in a categorical meta-analysis (i.e., estimating the pooled RR for the highest versus the lowest intake) or in a dose-response meta-analysis (i.e., estimating the pooled RR per one serving increment). Studies included in the categorical meta-analysis met the following eligibility criteria: a prospective cohort study or a nested case-control study; published in Korean or English; conducted among a human population; examining association of pickled vegetable or salted fish intake with gastric cancer incidence or mortality; and providing RRs and 95% CIs. Additional criteria for the studies included in the dose-response meta-analysis were as follows: providing more than two categories of exposure; indicating the number of cases and either the person-time or the total number of participants for each exposure category; and providing quantified exposure categories. When multiple studies reported on the same cohort, priority was given to studies addressing cancer incidence as an outcome, or reporting a higher number of cases, or to studies published more recently.

#### 2.2.3. Data Extraction and Quality Assessment

We extracted the following characteristics from the eligible studies: the first author’s last name, publication year, cohort, study region, recruitment period, follow-up period, exposure assessment method, dietary exposure, gastric cancer outcome, number of cases and total participants, and adjusted covariates. We assessed the quality of the cohort studies using the Strengthening the Reporting of Observational Studies in Epidemiology (STROBE) checklist for cohort studies [27].

#### 2.2.4. Meta-Analysis

We combined the RRs from the two large Korean cohort studies with those from previous cohort studies. We estimated the pooled RRs of gastric cancer per 40 g/day and 60 g/day increment in the intake of pickled vegetables and salted fish, respectively, and also estimated the pooled RR, comparing the highest to the lowest intake. All meta-analyses were performed using point estimate and standard error of the log RR, giving inverse-variance-weights to each study. We used either a random or a fixed effect model depending on the heterogeneity between the studies at a *P*-value < 0.10 [25]. The heterogeneity was assessed through the Cochran’s Q test, which quantifies the variation in study estimates due to heterogeneity [28]. In the presence of any heterogeneity, we conducted a sensitivity analysis by excluding either studies with gastric cancer mortality as outcome or those reporting deviating risk estimates. Test for non-linearity of the association was performed using restricted cubic splines [29]. We also conducted subgroup analyses by sex, gastric cancer outcome, publication year, follow-up period, and ethnicity and checked for heterogeneity through meta-regression [30]. The publication bias was assessed using Egger’s funnel plot [31].

All meta-analyses and illustrations of forest plots were performed using the R software (version 3.4.4; R Foundation for Statistical Computing, Vienna, Austria). Test for non-linearity was conducted using the SAS software, version 9.4 (SAS Institute Inc, Cary, North Carolina), and risk for bias assessment and the meta-regression were tested using the STATA software (version 15; Stata Corp, College Station, TX, USA). *P*-values of < 0.05 were considered statistically significant.

## 3. Results

### 3.1. The KoGES and the KMCC

In the KoGES, we identified 199 gastric cancer deaths during an average of 7.42 person-years and 201 gastric cancer deaths during an average of 7.43 person-years for the analyses of pickled vegetables and salted fish, respectively. Higher pickled vegetable consumers showed higher alcohol intake, pack-years, BMI, and energy intake (Table S2). Participants with a higher intake of salted fish showed similar characteristics (Table S3).

In the KMCC, we identified 81 cases of gastric cancer incidence during an average of 10.28 person-years for the analysis of pickled vegetables. In the analysis of salted fish, we identified 296 cases of gastric cancer incidence during an average of 12.86 person-years and 90 gastric cancer deaths during an average of 13.31 person-years. High pickled vegetable consumers were more likely to be current drinkers (Table S4), and high salted fish consumers tended to be current smokers (Table S5). The association between gastric cancer risk and the intake of pickled vegetables or salted fish was not clear in the KoGES and the KMCC (Table 1). The result was similar when we examined the association by sex or additionally adjusted for the fresh vegetable intake in the sensitivity analysis of pickled vegetable intake (Tables S6–S8).

### 3.2. Meta-Analysis

**Figure 1:**
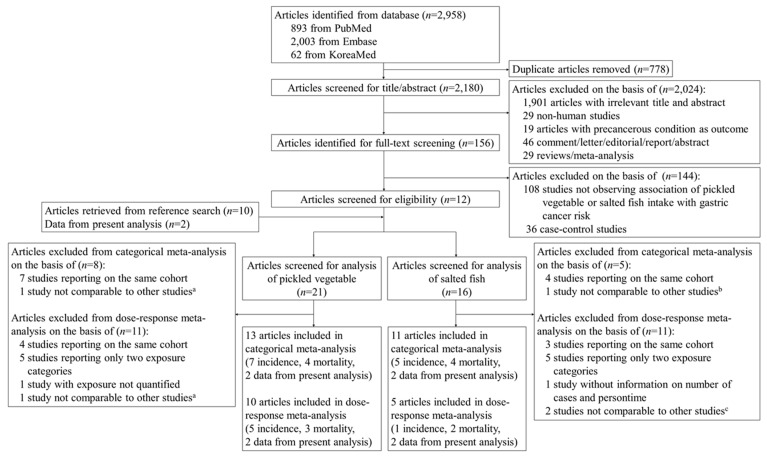
Flow diagram of study selection for meta-analysis. ^a^ Botterweck et al. [32]; ^b^ Kneller RW et al. [33]; ^c^ Sjodahl K et al. [34] and Kneller RW et al. [33].

We identified 2958 studies through a systematic search from the PubMed, Embase, and KoreaMed. After removing duplicates, 2180 studies were screened for title and abstract. 1901 studies with an irrelevant title and abstract, 29 non-human studies, 19 studies reporting a precancerous condition as the outcome, and 75 non-observational studies were excluded, leaving 156 studies eligible for full-text screening. During the full-text screening, we excluded 108 irrelevant studies and 36 case-control studies. We additionally retrieved 10 studies from reference lists and two studies from the present analysis, eventually including a total of 24 studies for eligibility screening. Among the 24 articles, 21 studies were eligible for the meta-analysis of pickled vegetables and 16 studies for that of salted fish (Figure 1). The characteristics of the included and excluded studies are summarized in Table 2 and Table S9, respectively.

#### 3.2.1. Pickled Vegetables

**Figure 2:**
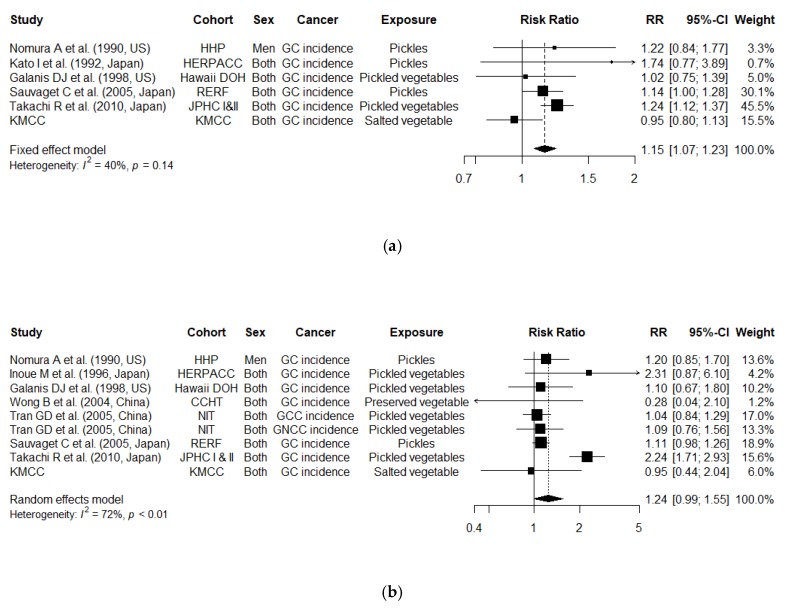
Study-specific and combined relative risks (95% confidence intervals) of gastric cancer incidence (**a**) per 40 g/day increment in pickled vegetable intake and (**b**) comparing the highest to the lowest intake of pickled vegetables; Abbreviation: GC, Gastric Cancer; GCC, Gastric Cardia Cancer; GNCC, Gastric Non Cardia Cancer; RR, Relative Risk; CI, Confidence Interval; CCHT, Changle County Helicobacter Trial; Hawaii DOH, Hawaii Department of Health Survey; HHP, Honolulu Heart Program; HERPACC, Hospital-based Epidemiologic Research Program at Aichi Cancer Center; JPHC, The Japan Public Health Center-based prospective Study; KMCC, Korean Multi-center Cancer Cohort; NIT, Linxian General Population Trial Cohort; RERF, Radiation Effects Research Foundation.

**Figure 3:**
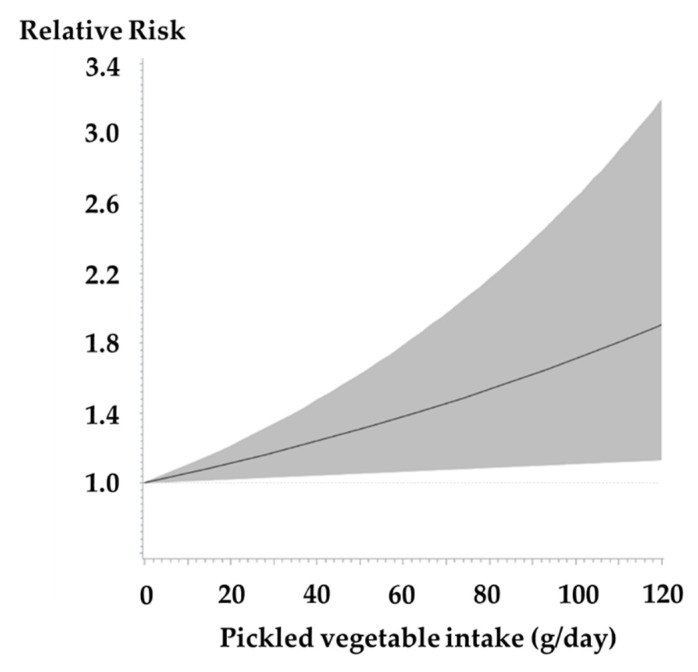
Continuous dose-response association between pickled vegetable intake and risk of gastric cancer incidence with restricted cubic splines; The solid line represents the estimated relative risk, and the shaded area represents 95% confidence intervals.

From a dose-response meta-analysis, we observed an increased risk of gastric cancer incidence for a 40 g/day increment in pickled vegetable intake (Figure 2a; *N* = 6 studies; combined RR, 1.15 [95% CI, 1.07–1.23]; *P* for heterogeneity = 0.14). The test for non-linearity using restricted cubic splines presented a linear association (Figure 3; *p* for non-linearity = 0.11). We additionally examined the risk for overall gastric cancer (incidence and mortality combined) and found an increased overall gastric cancer risk for a 40 g/day increment (Figure S1a; *N* = 10 studies; RR, 1.09 [95% CI, 1.00–1.18]; *P* for heterogeneity < 0.01). Decrease in heterogeneity was observed in a sensitivity analysis, where we excluded the KoGES, which reported gastric cancer mortality as outcome (RR, 1.14 [95% CI, 1.07–1.21]; *P* for heterogeneity = 0.38).

From a categorical meta-analysis, we observed a 1.24 times higher risk of gastric cancer incidence, comparing the highest to the lowest intake of pickled vegetables (Figure 2b; *N* = 8 studies; RR, 1.24 [95% CI, 0.99–1.55]; *P* for heterogeneity < 0.01). The heterogeneity was reduced when we excluded one study with a deviating risk estimate (RR, 1.10 [95% CI, 1.00–1.21]; *P* for heterogeneity = 0.70) [47]. The combined RR of the overall gastric cancer was 1.16 (*N* = 13 studies; 95% CI, 1.00–1.34; *P* for heterogeneity < 0.01), comparing the highest intake with the lowest intake (Figure S1b). Decrease in heterogeneity was observed when we excluded one study with a deviating risk estimate (RR, 1.08 [95% CI, 1.00–1.17]; *P* for heterogeneity = 0.86) [47].

#### 3.2.2. Salted Fish

**Figure 4:**
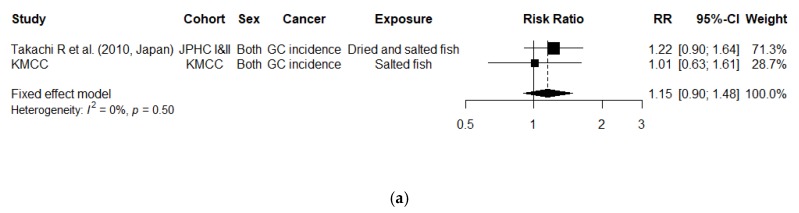

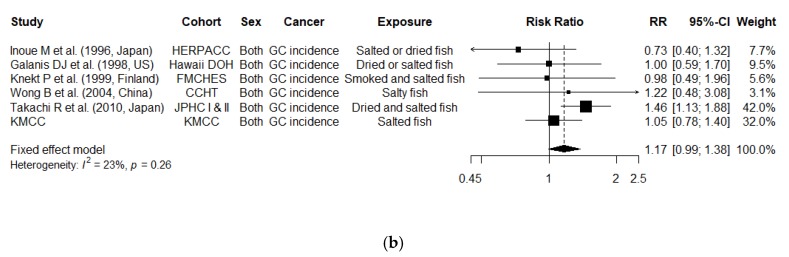
Study-specific and combined relative risks (95% confidence intervals) of gastric cancer incidence (**b**) per 60 g/day increment in salted fish intake and (**a**) comparing the highest to the lowest intake of salted fish; Abbreviation: GC, Gastric Cancer; RR, Relative Risk; CI, Confidence Interval; CCHT, Changle County Helicobacter Trial; FMCHES, Finnish Mobile Clinic Health Examination Survey; Hawaii DOH, Hawaii Department of Health Survey; HERPACC, Hospital-based Epidemiologic Research Program at Aichi Cancer Center; JPHC, The Japan Public Health Center-based prospective Study; KMCC, Korean Multi-center Cancer Cohort.

From the categorical meta-analysis, we found a marginally significant increase in the risk of gastric cancer incidence, comparing the highest to the lowest intake of salted fish (Figure 4b; *N* = 6 studies; RR, 1.17 [95% CI, 0.99–1.38]; *P* for heterogeneity = 0.26). When we further examined the association for the overall gastric cancer risk, the pooled RR was 1.10, but the association was not significant (Figure S2b; *N* = 11 studies; RR, 1.10 [95% CI, 0.98–1.23]; *P* for heterogeneity = 0.32).

There were not enough studies to conduct a dose-response meta-analysis of the gastric cancer incidence risk per 60 g/day increment in the salted fish intake. Only two studies were available, and the association was not significant. (Figure 4a; RR, 1.15 [95% CI, 0.90–1.48]; *P* for heterogeneity = 0.50). The result was similar for the analysis of overall gastric cancer risk (Figure S2a; *N* = 5 studies; RR, 1.09 [95% CI, 0.94–1.26]; *P* for heterogeneity = 0.89).

#### 3.2.3. Subgroup Analysis

We observed a significant heterogeneity by ethnicity in the pooled RR of overall gastric cancer per 40 g/day increment in pickled vegetable intake (*P* for heterogeneity = 0.002). No significant heterogeneity was observed by sex, outcome, publication year, and follow-up time (Table 3). We did not observe any significant heterogeneity by subgroups in the pooled RR of gastric cancer incidence (Table S10).

#### 3.2.4. Publication Bias

The Egger’s funnel plot asymmetry test failed to detect any small studies effect among the prospective cohort studies included in the meta-analyses (all *P*-values > 0.05). Therefore, we did not observe any publication bias (Figures S3–S6).

## 4. Discussion

In this systematic meta-analysis of prospective cohort studies, we found a significant association between increased gastric cancer incidence risk and high intake of pickled vegetables. The pooled risk of gastric cancer incidence was 1.15 times higher for a 40 g/day increment in pickled vegetable intake and 1.24 times higher, comparing the highest to the lowest intake. The result from our dose-response meta-analysis further strengthens previous meta-analyses, which showed an increased risk of gastric cancer, comparing the highest to the lowest intake of pickled vegetables and salted fish [12,13]. We also found a marginally significant increase in the risk of gastric cancer incidence for high salted fish intake.

Several mechanisms have been postulated in epidemiological studies regarding the association examined in our study. Pickled vegetables and salted fish are exogenous sources of sodium nitrates and nitrites, which react with amino acids in the stomach and form *N*-nitro compounds, known as chemical gastric carcinogens [49]. Epithelial damage induced by dietary salt intake is proposed as another possible mechanism of gastric carcinogenesis. High concentrations of sodium chloride induce mucosal damage, followed by cell proliferation as part of the repair process, sequentially increasing susceptibility to mutagenesis or carcinogenesis [9,50,51]. Previous animal studies have suggested that salt ingestion induces gastritis as well as intensification of gastric carcinogens [5,52,53]. Excessive cell proliferation in the gastric epithelium increases the potential for mutation, which may eventually result in intestinal metaplasia, one of the risk factors of gastric cancer. Vulnerability to gastric carcinogenesis is exacerbated through a synergistic effect with *H. pylori* infection. Cell proliferation induced by a high-salt diet facilitates *H. pylori* colonization, which sequentially promotes gastric inflammation, hypochlorhydria, Th1 and Th17 inflammation, and carcinogenesis [6,54,55]. In addition to previous animal studies reporting a combined effect of a high-salt diet and *H. pylori* infection [6,56], a recent prospective cohort study conducted in Colombia showed a significant association between dietary salt intake and an increase in epithelial damage, for which the degree was more prominent among participants infected with *H. pylori* infection [8].

Our findings from the two Korean cohort studies showed no associations for pickled vegetable and salted fish intake in relation to gastric cancer risk. Such incongruence may be attributable to skewed distribution of intake among the study participants. The intake amount of pickled vegetables for the reference group in the two Korean cohort studies corresponded to that of the intermediate or highest intake group in the other studies included in the meta-analysis [45,46,47]. Additionally, the high consumption of pickled vegetables in the Korean cohort studies was attributable to *kimchi*, a major Korean pickled vegetable dish prepared by salting and fermenting Napa cabbage. Although we adjusted for fresh vegetable intake in the sensitivity analysis, it is possible that high cabbage intake accompanied by high *kimchi* intake might have a mixed effect. Furthermore, salted fish intake in the Korean cohort studies might not have been sufficient enough to show a significant association with increased gastric cancer risk. The median values of each quantile of salted fish intake were smaller compared to those presented from a Japanese cohort study which showed a significant association [47].

The major strength of our study is that, to our knowledge, we were the first to examine the risk of gastric cancer mortality among the two large-scale Korean prospective cohort studies and further conduct a meta-analysis with literature studies. In our study, we excluded participants who died within three years from the baseline, thereby reducing the possibility of any subclinical disease influencing the diet at baseline. We also conducted both dose-response and categorical meta-analyses, thereby compensating for the possible loss of power due to solely conducting a categorical meta-analysis. Comparing the RR of the highest to the lowest intake category might neglect information regarding the intermediate intake of dietary exposure and reduce the power of association [57]. In addition, we included only prospective cohort studies in the meta-analysis. Although both case-control studies and cohort studies are susceptible to measurement error in regards to dietary assessment, the former is vulnerable to a difference in the recall of diet or selection bias [58].

Our study has several limitations. First, the inability to conduct a periodic evaluation of the dietary intake might not adequately reflect possible changes in diet over time [59]. Second, we could not estimate the pooled RRs by geographical region due to insufficient number of Western prospective cohort studies. Lastly, we could not observe whether *H. pylori* infection, a class I carcinogen of gastric cancer, affected the association of gastric cancer and the intake of pickled vegetables and salted fish due to insufficient information provided from the participants in both cohorts.

## 5. Conclusions

Our meta-analysis results provided evidence that a high intake of pickled vegetables and salted fish is significantly associated with increased gastric cancer risk. Although further prospective cohort studies are warranted, reduction in pickled vegetable and salted fish may be an appropriate public health intervention aimed at reducing the risk of gastric cancer.

## Figures and Tables

**Table 1:** Relative risks (95% confidence intervals) of gastric cancer risk according to pickled vegetable or salted fish intake in the Korean cohort studies.

**Outcome**			**Pickled Vegetable Intake**					***P* for Trend**	**Per 40 g/day Increment**
			**Quintile 1**	**Quintile 2**	**Quintile 3**	**Quintile 4**	**Quintile 5**		
**Incidence**									
	**KMCC**							
		Case no.	15	15	18	21	12		81
		Person-years	9153.04	8983.91	9774.26	11020.96	7474.97		46407.13
		Model 1 ^a^	1.00 (reference)	0.96 (0.47–1.97)	1.19 (0.60–2.37)	1.09 (0.56–2.12)	0.93 (0.44–1.99)	0.98	0.95 (0.80–1.13)
		Model 2 ^b^	1.00 (reference)	0.99 (0.48–2.03)	1.21 (0.60–2.40)	1.10 (0.56–2.15)	0.95 (0.44–2.04)	0.97	0.95 (0.80–1.13)
**Mortality**									
	**KoGES**							
		Case no.	35	34	31	49	50		199
		Person-years	280471.68	282869.54	283603.52	289748.48	314506.54		1451199.76
		Model 1 ^c^	1.00 (reference)	0.84 (0.52–1.35)	0.79 (0.49–1.29)	1.14 (0.73–1.77)	0.89 (0.57–1.40)	0.99	0.99 (0.95–1.03)
		Model 2 ^d^	1.00 (reference)	0.83 (0.52–1.33)	0.79 (0.49–1.28)	1.14 (0.73–1.77)	0.85 (0.54–1.34)	0.84	0.99 (0.95–1.03)
**Outcome**			**Salted Fish Intake**					***P* for Trend**	**Per 60 g/day Increment**
			**Tertile 1**		**Tertile 2**	**Tertile 3**			
**Incidence**									
	**KMCC**								
		Case no.	88		113	95			296
		Person-years	45481.01		53529.26	46630.12			145640.40
		Model 1^a^	1.00 (reference)		1.15 (0.87–1.53)	1.08 (0.80–1.44)		0.85	1.10 (0.70–1.73)
		Model 2^b^	1.00 (reference)		1.05 (0.78–1.40)	1.03 (0.77–1.38)		0.94	1.01 (0.63–1.61)
**Mortality**									
	**KMCC**								
		Case no.	22		42	26			90
		Person-years	45690.51		53829.00	46943.62			146463.13
		Model 1 ^a^	1.00 (reference)		1.74 (1.03–1.93)	1.26 (0.71–1.24)		0.93	1.50 (0.69–3.23)
		Model 2 ^b^	1.00 (reference)		1.39 (0.81–1.38)	1.12 (0.63–1.00)		0.91	1.22 (0.54–2.78)
	**KoGES**								
		Case no.	72		62	67			201
		Person-years	491079.82		465063.62	502876.12			1459019.56
		Model 1 ^c^	1.00 (reference)		1.03 (0.73–1.45)	0.90 (0.64–1.27)		0.48	1.89 (0.37–9.70)
		Model 2 ^d^	1.00 (reference)		1.03 (0.73–1.45)	0.86 (0.61–1.22)		0.32	1.40 (0.26–7.52)
	**Pooled**								
		MV adjusted	1.00 (reference)		1.12 (0.84–1.50)	0.92 (0.68–1.24)		0.80	1.25 (0.60–2.62)

Abbreviations: KMCC, Korean Multi-center Cancer Cohort; KoGES, Korean Genome Epidemiology Study; CAVAS, Cardiovascular Disease Association Study; HEXA, Health Examinee; BMI, Body Mass Index; MV, Multivariate. ^a^ Model 1: Stratified by age (20 to < 50, 50 to < 60, 60 to < 70, and 70+ years) and adjusted for age at baseline (continuous, years) and sex. ^b^ Model 2: Model 1 further adjusted for survey year (continuous, year), BMI (10 to < 18.5, 18.5 to < 23, 23 to < 25, 25 to < 30, and 30+ kg/m^2^ for analysis of incidence and 10 to < 23, 23 to < 25, and 25+ kg/m^2^ for analysis of mortality), smoking status (never smoker, past smoker, and current smoker), and alcohol drinking frequency (never, 0 < to < 1 time per week, 1 time per week to < 3 times per week, 3 times per week to <1 time per day, and 1+ time per day) ^c^ Model 1: Stratified by age (40 to < 50, 50 to < 60, and 60+ years) and study cohort (the Ansan and Ansung study, the CAVAS, and the HEXA study) and further adjusted for age at baseline (continuous, year), sex, and total energy intake (continuous, kcal/day).^d^ Model 2: Model 1 further adjusted for survey year (continuous, year), BMI (10 to < 18.5, 18.5 to < 23, 23 to < 25, 25 to < 30, and 30+ kg/m^2^), smoking status (0, 0 < to < 10, 10 to < 20, and 20+ pack-years), and alcohol intake (0, 0 < to < 5, 5 to < 15, 15 to < 30, and 30+ g/day).

**Table 2:** Characteristics of studies included in the meta-analysis.

First Author, Year	Study	Country	Recruitment Period/Follow-up Period	Exposure Assessment	Exposure	Outcome	Cases/total Participants	Adjusted Variables
Nomura A et al., 1990 [35] ^a^	Honolulu Hearth Program (HHP)	US (Japanese ancestry)	1965–1968/ Average 10.6 years	20-item FFQ	Pickles	Incidence	150/7990	Age
Kneller RW et al., 1991 [33] ^b^	Lutheran Brotherhood Insurance Society (LBS)	US	1966/20 years	35-item FFQ	Salted fish	Mortality	72/17,633	Year of birth and current cigarette smoking
Kato I et al., 1992 [36] ^c^	Hospital-based Epidemiologic Research Program at Aichi Cancer Center (HERPACC)	Japan	1985–1989/4.4 years	10-item questionnaire	Pickles	Incidence	45/3914	Sex, age, and residence
Kato I et al., 1992 [37] ^a^	Higashi-Kamo Cohort	Japan	1985/ Until 1991	25-item questionnaire	Pickles	Mortality	57/9753	Age and sex
Inoue M et al., 1996 [38] ^b^	Hospital-based Epidemiologic Research Program at Aichi Cancer Center (HERPACC)	Japan	1985–1989/ Until 1995	FFQ	Pickled vegetables, salted or dried fish	Incidence	69/5373	Gender and age
Galanis DJ et al., 1998 [39] ^a^	Hawaii Department of Health Survey	US (Japanese ancestry)	1975–1980/14.8 years	FFQ	Pickled vegetables, dried or salted fish	Incidence	108/11,907	Age, years of education, Japanese place of birth, and gender (in combined analysis)
Knekt P et al., 1999 [40] ^b^	Finnish Mobile Clinic Health Examination Survey (FMCHES)	Finland	1966–1972/24 years	Dietary history interview	Smoked and salted fish	Incidence	68/9985	Sex, age, municipality, smoking, and energy intake
Ngoan LT et al., 2002 [41] ^a^	Miyako Study	Japan	1986–1989/ Until 1999	25-item FFQ	Pickled food, processed fish	Mortality	59/7483	Age, sex, smoking, processed meat, liver, cooking or salad oil, suimono
Khan MMH et al., 2004 [42] ^b^	Hokkaido Cohort	Japan	1984–1985/ Until 2002	FFQ	Japanese pickle, salty fish	Mortality	51/3158	Men: Age and smoking Women: Age, health status, health education, health screening, and smoking
Tsugane S et al., 2004 [43] ^d^	The Japan Public Health Center-based prospective Study (JPHC I and II)	Japan	1990/ Until 2001	FFQ	Pickled vegetables, dried or salted fish	Incidence	486/39,065	Age in 1990, cigarette smoking, and fruit and non-green-yellow vegetable intake, quartile categories of salt intake, and stratified by PHC area
Wong B et al., 2004 [44] ^b^	Changle County Helicobacter Trial (CCHT)	China	1994/ Until 2002	FFQ	Preserved vegetables, salty fish	Incidence	18/1630	N/A
Sauvaget C et al., 2005 [45] ^a^	Radiation Effects Research Foundation (RERF)	Japan	Men: 1978–1980; Women: 19781981–/20 years	22-item FFQ	Pickles	Incidence	1270/ 38,576	Age, sex, city, radiation dose, sex-specific smoking habit and education
Iso H et al., 2007 [46] ^a^	The Japan Collaborative Cohort Study (JACC)	Japan	1988–1990/ Until 2003	FFQ	Pickles, dried or salted fish	Mortality	1076/ 101,190	Age and area of study
Takachi R et al., 2010 [47] ^a^	The Japan Public Health Center-based prospective Study (JPHC I and II)	Japan	1990 (JPHC I); 1993 (JPHC II)/ Until 2004	138-item FFQ	Pickled vegetables, dried and salted fish	Incidence	876/77,500	Sex, age, BMI, smoking status, alcohol consumption, physical activity in metabolic equivalent task-hours/d, and quintiles of energy, potassium, and calcium
Tran GD et al., 2005 [48] ^b^	Linxian General Population Trial Cohort (NIT)	China	1984/ Until 2001	9-item FFQ	Pickled vegetables	Incidence (gastric cardia cancer)	1089/29,584	Age and gender
						Incidence (gastric non-cardia cancer)	363/29,584	

Abbreviation: FFQ, Food Frequency Questionnaire. ^a^ Studies included in both dose-response and categorical meta-analysis. ^b^ Studies included in categorical meta-analysis only. ^c^ Studies included in dose-response meta-analysis only. ^d^ Study included instead of Takachi R et al. [47] when conducting subgroup analysis by sex.

**Table 3:** Stratified analysis of pickled vegetable or salted fish intake and overall gastric cancer risk (incidence and mortality combined).

Subgroup			No. of Studies	RR (95% CI)		Q Test, *p*-Value	*P* for Difference
				Fixed-Effects Model	Random-Effects Model		
**Pickled vegetable intake**							
	**Dose-response analysis (per 40 g/day increment)**						
		**Sex**					0.93
		Men	6	1.00 (0.95, 1.05)	1.04 (0.94, 1.15)	0.19	
		Women	5	1.00 (0.94, 1.07)	1.00 (0.94, 1.07)	0.95	
		**Outcome**					0.24
		Incidence	6	1.15 (1.07, 1.23)	1.13 (1.02, 1.25)	0.14	
		Mortality	4	1.00 (0.96, 1.04)	1.00 (0.96, 1.04)	0.55	
		**Publication year**					0.91
		Before 2000	4	1.10 (0.89, 1.36)	1.10 (0.89, 1.36)	0.47	
		Since 2000	6	1.03 (1.00, 1.07)	1.09 (0.99, 1.19)	0.002	
		**Follow-up time**					0.67
		<15 years	9	1.03 (0.99, 1.06)	1.08 (0.98, 1.19)	0.01	
		≥15 years	1	1.14 (1.00, 1.28)	1.14 (1.00, 1.28)	-	
		**Ethnicity**					0.002
		Korea	2	0.99 (0.95, 1.03)	0.99 (0.95, 1.03)	0.70	
		Japan	8	1.17 (1.10, 1.25)	1.17 (1.10, 1.25)	0.77	
	**High versus low analysis**						
		**Sex**					0.73
		Men	8	1.10 (0.96, 1.27)	1.10 (0.96, 1.27)	0.64	
		Women	6	1.06 (0.85, 1.32)	1.06 (0.85, 1.32)	0.98	
		**Outcome**					0.25
		Incidence	8	1.19 (1.09, 1.31)	1.24 (0.99, 1.55)	0.001	
		Mortality	5	1.04 (0.90, 1.20)	1.04 (0.90, 1.20)	0.75	
		**Publication year**					0.99
		Before 2000	4	1.14 (0.88, 1.47)	1.14 (0.85, 1.52)	0.33	
		Since 2000	9	1.15 (1.06, 1.24)	1.16 (0.97, 1.37)	0.001	
		**Follow-up time**					0.54
		<15 years	10	1.22 (1.09, 1.37)	1.18 (0.93, 1.50)	0.001	
		≥15 years	3	1.09 (0.99, 1.21)	1.09 (0.99, 1.21)	0.97	
		**Ethnicity**					0.19
		Korea, China	4	1.01 (0.86, 1.19)	1.01 (0.86, 1.19)	0.67	
		Japan	9	1.19 (1.09, 1.30)	1.24 (1.02, 1.51)	0.001	
**Salted fish intake**							
	**Dose-response analysis (per 60 g/day increment)**						
		**Sex**					0.22
		Men	4	1.21 (1.00, 1.45)	1.21 (1.00, 1.45)	0.97	
		Women	4	0.94 (0.70, 1.27)	0.94 (0.70, 1.27)	0.82	
		**Outcome**					0.62
		Incidence	2	1.15 (0.90, 1.48)	1.15 (0.90, 1.48)	0.50	
		Mortality	4	1.06 (0.89, 1.27)	1.06 (0.89, 1.27)	0.90	
		**Publication year**					-
		Before 2000	0	-	-	-	
		Since 2000	5	1.09 (0.94, 1.26)	1.09 (0.94, 1.26)	0.89	
		**Follow-up time**					-
		<15 years	5	1.09 (0.94, 1.26)	1.09 (0.94, 1.26)	0.89	
		≥15 years	0	-	-	-	
		**Ethnicity**					0.82
		Korea	2	1.03 (0.66, 1.62)	1.03 (0.66, 1.62)	0.72	
		Japan	3	1.09 (0.94, 1.28)	1.09 (0.94, 1.28)	0.68	
	**High versus low analysis**						
		**Sex**					0.46
		Men	7	1.10 (0.94, 1.28)	1.12 (0.90, 1.39)	0.15	
		Women	6	0.99 (0.79, 1.24)	0.99 (0.79, 1.24)	0.89	
		**Outcome**					0.59
		Incidence	6	1.17 (1.00, 1.38)	1.13 (0.92, 1.39)	0.25	
		Mortality	6	1.08 (0.94, 1.25)	1.08 (0.94, 1.25)	0.73	
		**Publication year**					0.88
		Before 2000	4	1.05 (0.78, 1.42)	1.06 (0.73, 1.54)	0.21	
		Since 2000	7	1.13 (1.01, 1.27)	1.13 (1.01, 1.27)	0.55	
		**Follow-up time**					0.50
		<15 years	8	1.11 (0.99, 1.24)	1.10 (0.98, 1.25)	0.38	
		≥15 years	3	1.24 (0.86, 1.77)	1.24 (0.86, 1.77)	0.43	
		**Ethnicity**					0.86
		Korea, China, Europe	5	1.10 (0.90, 1.34)	1.10 (0.90, 1.34)	0.55	
		Japan	6	1.13 (0.99, 1.29)	1.11 (0.95, 1.30)	0.29	

Abbreviations: RR, Relative Risk; CI, Confidence Interval.

## Supplementary Materials

The following are available online at https://www.mdpi.com/2072-6694/12/4/996/s1, Figure S1: Study-specific and combined relative risks (95% confidence intervals) of overall gastric cancer risk (incidence and mortality combined) (a) per 40 g/day increment in pickled vegetable intake and (b) comparing the highest to the lowest intake of pickled vegetables; Figure S2: Study-specific and combined relative risks (95% confidence intervals) of overall gastric cancer risk (incidence and mortality combined) (a) per 60 g/day increment in salted fish intake and (b) comparing the highest to the lowest intake of salted fish; Figure S3: Funnel plot for the studies included in (a) dose-response meta-analysis and (b) categorical meta-analysis of pickled vegetable intake with gastric cancer incidence; Figure S4: Funnel plot for the studies included in (a) dose-response meta-analysis^a^ and (b) categorical meta-analysis of salted fish intake with gastric cancer incidence; Figure S5: Funnel plot for the studies included in (a) dose-response meta-analysis and (b) categorical meta-analysis of pickled vegetable intake with gastric cancer risk (incidence and mortality combined); Figure S6: Funnel plot for the studies included in (a) dose-response meta-analysis and (b) categorical meta-analysis of salted fish intake with gastric cancer risk (incidence and mortality combined); Table S1: Terminology used for article search on the literature database; Table S2: Baseline characteristics of participants from the Korean Genome Epidemiology Study according to pickled vegetable intake; Table S3: Baseline characteristics of participants from the Korean Genome Epidemiology Study according to salted fish intake, Table S4: Baseline characteristics of the Korean Multi-center Cancer Cohort study participants according to pickled vegetable intake; Table S5: Baseline characteristics of the Korean Multi-center Cancer Cohort study participants according to salted fish intake; Table S6: Relative risks (95% confidence intervals) of gastric cancer risk by sex according to pickled vegetable intake in the Korean cohort studies; Table S7: Relative risks (95% confidence intervals) of gastric cancer risk by sex according to salted fish intake in the Korean cohort studies; Table S8: Sensitivity analysis of gastric cancer risk according to pickled vegetable intake in the Korean cohort studies; Table S9: Characteristics of studies excluded from the meta-analysis, Table S10: Stratified analysis of pickled vegetable or salted fish intake with the risk of gastric cancer incidence.
