# Supplementary material for: Pickled Vegetable and Salted Fish Intake and the Risk of Gastric Cancer: Two Prospective Cohort Studies and a Meta-Analysis
Source: Cancers (Basel). 2020 Apr 17;12(4):996. doi: 10.3390/cancers12040996 (PMC7225928; doi:10.3390/cancers12040996)
Supplement: Supplementary file 1 [file cancers-12-00996-s001.pdf]

Review

Pickled Vegetable and Salted Fish Intake and the Risk of Gastric Cancer: Two Prospective Cohort Studies and a Meta-Analysis

Jin Young Yoo, Hyun Jeong Cho, Sungji Moon, Jeoungbin Choi, Sangjun Lee, Choonghyun Ahn, Keun-Young Yoo, Inah Kim, Kwang-Pil Ko, Jung Eun Lee and Sue K. Park

Table S1. Terminology used for article search on the literature database.

Database	Search term	Items found
**PubMed**		
#17	Search ((#10 AND #11) OR (#10 AND #12) OR (#10 AND #13) OR (#10 AND #14)) Filters: Humans; English	893
#16	Search ((#10 AND #11) OR (#10 AND #12) OR (#10 AND #13) OR (#10 AND #14)) Filters: Humans	1049
#15	Search ((#10 AND #11) OR (#10 AND #12) OR (#10 AND #13) OR (#10 AND #14))	1470
#14	Search (gastric cancer mortality[Title/Abstract] OR gastric cancer death[Title/Abstract])	227
#13	Search (gastric cancer[Title/Abstract] OR gastric cancer[MeSH Terms] OR gastric neoplasm[MeSH Terms])	109401
#12	Search (stomach cancer mortality[Title/Abstract] OR stomach cancer death[Title/Abstract])	172
#11	Search (stomach cancer[Title/Abstract] OR stomach cancer[MeSH Terms] OR stomach neoplasm[MeSH Terms])	95671
#10	Search (#1 OR #2 OR #3 OR #4 OR #5 OR #6 OR #7 OR #8 OR #9)	317667
#9	Search dried fish[Title/Abstract]	168
#8	Search salted fish[Title/Abstract]	217
#7	Search pickled vegetable[Title/Abstract]	33
#6	Search salted vegetable[Title/Abstract]	219
#5	Search food preserved by salting[Title/Abstract]	20
#4	Search high-salt food[Title/Abstract]	17
#3	Search preserved food[Title/Abstract]	102
#2	Search salted food[Title/Abstract]	70
#1	Search diet[Title/Abstract]	317014
**Embase**		
#25	(#10 AND #13) OR (#10 AND #16) OR (#10 AND #19) OR (#10 AND #22) AND [humans]/lim AND [english]/lim	2003
#24	(#10 AND #13) OR (#10 AND #16) OR (#10 AND #19) OR (#10 AND #22) AND [humans]/lim	2277
#23	(#10 AND #13) OR (#10 AND #16) OR (#10 AND #19) OR (#10 AND #22)	2808
#22	#20 OR #21	306
#21	'gastric cancer death'/exp OR 'gastric cancer death':ab,ti	57
#20	'gastric cancer mortality'/exp OR 'gastric cancer mortality':ab,ti	260
#19	#17 OR #18	187
#18	'stomach cancer death'/exp OR 'stomach cancer death':ab,ti	24
#17	'stomach cancer mortality'/exp OR 'stomach cancer mortality':ab,ti	169
#16	#14 OR #15	133356
#15	'gastric neoplasm'/exp OR 'gastric neoplasm':ab,ti	737
#14	'gastric cancer'/exp OR 'gastric cancer':ab,ti	133095
#13	#11 OR #12	157584
#12	'stomach neoplasm'/exp OR 'stomach neoplasm':ab,ti	156496
#11	'stomach cancer'/exp OR 'stomach cancer':ab,ti	118501

#10	#1 OR #2 OR #3 OR #4 OR #5 OR #6 OR #7 OR #8 OR #9	540226
#9	'dried fish'/exp OR 'dried fish':ab,ti	372
#8	'salted fish'/exp OR 'salted fish':ab,ti	249
#7	'pickled vegetable'/exp OR 'pickled vegetable':ab,ti	40
#6	'salted vegetable'/exp OR 'salted vegetable':ab,ti	9
#5	'food preserved by salting'/exp OR 'food preserved by 'salting':ab,ti	1
#4	'high-salt food'/exp OR 'high-salt food':ab,ti	25
#3	'preserved food'/exp OR 'preserved food':ab,ti	1697
#2	'salted food'/exp OR 'salted food':ab,ti	97
#1	'diet'/exp OR 'diet':ab,ti	538429
**KoreaMed**		
#24	#19 OR #20 OR #21 OR #22 Filters: Humans;	62
#23	#19 OR #20 OR #21 OR #22	67
#22	(#10 AND #18)	1
#21	(#10 AND #15)	57
#20	(#10 AND #14)	1
#19	(#10 AND #11)	13
#18	#16 OR #17	9
#17	(TIAB:"gastric cancer death") OR MH:"gastric cancer death"	1
#16	(TIAB:"gastric cancer mortality") OR MH:"gastric cancer mortality"	9
#15	(TIAB:"gastric cancer") OR MH:"gastric cancer"	2946
#14	#12 OR #13	3
#13	(TIAB:"stomach cancer death") OR MH:"stomach cancer death"	2
#12	(TIAB:"stomach cancer mortality") OR MH:"stomach cancer mortality"	1
#11	(TIAB:"stomach cancer") OR MH:"stomach cancer"	688
#10	#1 OR #2 OR #3 OR #4 OR #5 OR #6 OR #7 OR #8 OR #9	3573
#9	TIAB:"dried fish"	6
#8	TIAB:"salted fish"	8
#7	TIAB:"pickled vegetable"	6
#6	TIAB:"salted vegetable"	13
#5	TIAB:"food preserved by salting"	0
#4	TIAB:"high-salt food"	1
#3	TIAB:"preserved food"	5
#2	TIAB:"salted food"	9
#1	TIAB:"diet"	3543

Table S2. Baseline characteristics of participants from the Korean Genome Epidemiology Study according to pickled vegetable intake.

Characteristic	Pickled vegetable intake				
	Quintile 1	Quintile 2	Quintile 3	Quintile 4	Quintile 5
**Median intake, g/day (range)**	31.44 (0–56.94)	80.09 (56.94–103.93)	136.46 (103.93–159.14)	183.21 (159.14–230.19)	303.95 (230.19–1215.00)
**No. of participants**	39136	39103	39134	39132	39119
**Age (years), mean (SD)**	53.66 (8.73)	53.89 (8.88)	53.38 (8.62)	53.63 (8.56)	53.75 (8.72)
**Sex, *N* (%)**					
Men	11632 (29.72)	12626 (32.29)	13658 (34.90)	15282 (39.05)	16540 (42.28)
Women	27504 (70.28)	26477 (67.71)	25476 (65.10)	23850 (60.95)	22579 (57.72)
**Study cohort, *N* (%)**					
Ansan and Ansung Study	811 (2.07)	1444 (3.69)	1596 (4.08)	2136 (5.46)	2591 (6.62)
CAVAS	4578 (11.70)	5586 (14.29)	4723 (12.07)	4921 (12.58)	6524 (16.68)
HEXA Study	33747 (86.23)	32073 (82.02)	32815 (83.85)	32075 (81.97)	30004 (76.70)
**Alcohol intake (g/day), mean (SD)**	5.71 (17.94)	6.68 (22.40)	6.95 (18.87)	7.93 (25.77)	9.07 (23.89)
**Smoking (pack-years), mean (SD)**	5.04 (12.64)	5.56 (12.79)	5.91 (13.11)	6.70 (13.78)	7.33 (14.33)
**BMI (kg/m^2), mean (SD)**	23.81 (2.93)	23.91 (2.93)	24.00 (2.94)	24.13 (2.95)	24.36 (2.98)
**Energy intake (kcal/day), mean (SD)**	1542.92 (478.99)	1659.09 (485.69)	1721.68 (490.14)	1824.48 (491.66)	1993.64 (582.48)

Abbreviations: BMI, Body Mass Index; SD, Standard Deviation; CAVAS, Cardiovascular Disease Association Study; HEXA, Health Examinee.

Table S3. Baseline characteristics of participants from the Korean Genome Epidemiology Study according to salted fish intake

Characteristic	Salted fish intake		
	**Tertile 1**	**Tertile 2**	**Tertile 3**
**Median intake, g/day (range)**	0 (0–0.37)	0.75 (0.40–1.51)	2.89 (1.54–86.48)
**No. of participants**	66391	63395	66598
**Age (years), mean (SD)**	53.75 (8.99)	53.09 (8.53)	54.09 (8.56)
**Sex, *N* (%)**			
Men	22189 (33.42)	22873 (36.08)	24876 (37.35)
Women	44202 (66.58)	40522 (63.92)	41722 (62.65)
**Study cohort, *N* (%)**			
Ansan and Ansung Study	1786 (2.69)	2798 (4.41)	4140 (6.22)
CAVAS	11919 (17.95)	6857 (10.82)	7622 (11.44)
HEXA Study	52686 (79.36)	53740 (84.77)	54836 (82.34)
**Alcohol intake (g/day), mean (SD)**	5.82 (20.10)	7.03 (18.16)	8.91 (26.52)
**Smoking (pack-years), mean (SD)**	5.39 (12.79)	5.93 (12.88)	6.98 (14.32)
**BMI (kg/m²), mean (SD)**	23.91 (2.95)	24.00 (2.93)	24.21 (2.96)
**Energy intake (kcal/day), mean (SD)**	1607.40 (479.90)	1710.89 (488.30)	1926.92 (566.94)

Abbreviations: BMI, Body Mass Index; SD, Standard Deviation; CAVAS, Cardiovascular Disease Association Study; HEXA, Health Examinee.

Table S4. Baseline characteristics of the Korean Multi-center Cancer Cohort study participants according to pickled vegetable intake.

Characteristic	Pickled vegetable intake				
	**Quintile 1**	**Quintile 2**	**Quintile 3**	**Quintile 4**	**Quintile 5**
**Median intake, g/day (range)**	41.33 (0–62.86)	80.00 (68.57–84.65)	94.29 (85.71–114.29)	160.00 (120.00–160.00)	165.71 (161.33–240.00)
**No. of participants**	894	875	943	1069	732
**Age (years), mean (SD)**	57.71 (12.86)	59.88 (11.86)	56.28 (11.89)	58.64 (11.06)	57.88 (11.53)
**Sex, *N* (%)**					
Men	349 (39.04)	299 (34.17)	376 (39.87)	415 (38.82)	294 (40.16)
Women	545 (60.96)	576 (65.83)	567 (60.13)	654 (61.18)	438 (59.84)
**Alcohol drinking, *N* (%)**					
Never	546 (61.07)	577 (65.94)	571 (60.55)	626 (58.56)	428 (58.47)
0< to <1 time per week	91 (10.18)	81 (9.26)	83 (8.80)	95 (8.89)	65 (8.88)
1 time per week to <3 times per week	131 (14.65)	93 (10.63)	122 (12.94)	151 (14.13)	94 (12.84)
3 times per week to <1 time per day	63 (7.05)	45 (5.14)	71 (7.53)	65 (6.08)	60 (8.20)
1+ time per day	63 (7.05)	79 (9.03)	96 (10.18)	132 (12.35)	85 (11.61)
**Smoking status, *N* (%)**					
Never smoker	556 (62.68)	581 (66.48)	621 (65.99)	678 (63.66)	484 (66.21)
Past smoker	133 (14.99)	139 (15.90)	146 (15.52)	171 (16.06)	105 (14.36)
Current smoker	198 (22.32)	154 (17.62)	174 (18.49)	216 (20.28)	142 (19.43)
**BMI (kg/m^2), mean (SD)**	24.13 (3.28)	24.05 (3.09)	24.23 (3.17)	24.43 (3.17)	24.34 (3.25)

Abbreviations: BMI, Body Mass Index; SD, Standard Deviation.

Table S5. Baseline characteristics of the Korean Multi-center Cancer Cohort study participants according to salted fish intake.

Characteristic	Salted fish intake		
	Tertile 1	Tertile 2	Tertile 3
**Median intake, g/day (range)**	0 (0–1.99)	6.98 (4.98–6.98)	30.00 (8.57–120.00)
**No. of participants**	3762	3849	3711
**Age (years), mean (SD)**	58.92 (11.66)	56.96 (11.15)	56.25 (11.02)
**Sex, *N* (%)**			
Men	1338 (35.57)	1460 (37.93)	1628 (43.87)
Women	2424 (64.43)	2389 (62.07)	2083 (56.13)
**Alcohol drinking, *N* (%)**			
Never	2616 (69.54)	2698 (70.10)	2282 (61.49)
0< to <1 time per week	298 (7.92)	269 (6.99)	236 (6.36)
1 time per week to <3 times per week	346 (9.20)	393 (10.21)	487 (13.12)
3 times per week to <1 time per day	179 (4.76)	185 (4.81)	261 (7.03)
1+ time per day	323 (8.59)	304 (7.90)	445 (11.99)
**Smoking status, *N* (%)**			
Never smoker	2431 (64.83)	2454 (63.94)	2260 (61.13)
Past smoker	463 (12.35)	376 (9.80)	449 (12.14)
Current smoker	856 (22.83)	1008 (26.26)	998 (26.72)
**BMI (kg/m^2), mean (SD)**	23.69 (3.24)	23.61 (3.27)	24.05 (3.22)

Abbreviations: BMI, Body Mass Index; SD, Standard Deviation.

Table S6. Relative risks (95% confidence intervals) of gastric cancer risk by sex according to pickled vegetable intake in the Korean cohort studies.

Outcome	Pickled vegetable intake					*P* for trend	Per increment of 40 g/day
	Quintile 1	Quintile 2	Quintile 3	Quintile 4	Quintile 5		
**Men**							
**Incidence**							
**KMCC**							
Median, g/day (range)	40.00 (0–62.86)	80.00 (68.57–84.65)	94.29 (85.71–114.29)	160.00 (120.00–160.00)	174.29 (161.33–240.00)		
Case no.	9	7	14	12	7		49
Person-years	3387.60	2928.41	3695.53	4119.03	2885.74		17016.32
Model 1[a]	1.00 (reference)	0.79 (0.29–2.13)	1.45 (0.63–3.37)	0.99 (0.42–2.36)	0.84 (0.31–2.27)	0.78	0.92 (0.74–1.15)
Model 2[b]	1.00 (reference)	0.82 (0.30–2.20)	1.46 (0.63–3.41)	1.02 (0.43–2.46)	0.86 (0.32–2.34)	0.83	0.92 (0.74–1.15)
**Mortality**							
**KoGES**							
Median, g/day (range)	32.27 (0–56.94)	80.36 (56.94–103.93)	134.97 (103.93–159.12)	184.25 (159.14–230.19)	305.18 (230.19–1215.00)		
Case no.	21	21	19	34	35		130
Person-years	83930.47	90997.98	99410.33	113236.49	132516.96		520092.24
Model 1[c]	1.00 (reference)	0.84 (0.46–1.54)	0.75 (0.40–1.39)	1.16 (0.67–2.01)	0.88 (0.51–1.54)	>0.99	0.99 (0.94–1.04)
Model 2[d]	1.00 (reference)	0.82 (0.45–1.50)	0.74 (0.40–1.39)	1.16 (0.67–2.01)	0.84 (0.48–1.48)	0.88	0.98 (0.93–1.04)
**Women**							
**Incidence**							
**KMCC**							
Median, g/day (range)	41.33 (0–62.86)	80.00 (68.57–84.65)	94.29 (85.71–114.29)	160.00 (120.00–160.00)	165.71 (161.33–240.00)		
Case no.	6	8	4	9	5		32
Person-years	5765.44	6055.50	6078.73	6901.92	4589.22		29390.81
Model 1[a]	1.00 (reference)	1.20 (0.42–3.47)	0.73 (0.21–2.58)	1.27 (0.45–3.58)	1.10 (0.34–3.63)	0.67	1.01 (0.77–1.33)
Model 2[b]	1.00 (reference)	1.21 (0.42–3.50)	0.72 (0.20–2.58)	1.27 (0.45–3.61)	1.11 (0.33–3.69)	0.67	1.01 (0.77–1.34)
**Mortality**							
**KoGES**							
Median, g/day (range)	31.15 (0–56.94)	79.93 (56.94–103.91)	138.29 (103.93–159.14)	182.73 (159.14–230.19)	302.82 (230.19–1215.00)		
Case no.	14	13	12	15	15		69
Person-years	196541.21	191871.55	184193.19	176511.98	181989.58		931107.52
Model 1[c]	1.00 (reference)	0.85 (0.40–1.82)	0.89 (0.41–1.93)	1.11 (0.53–2.33)	0.93 (0.43–1.98)	0.95	1.01 (0.94–1.08)

Model 2[d]	1.00 (reference)	0.83 (0.39–1.78)	0.86 (0.39–1.86)	1.07 (0.51–2.24)	0.83 (0.39–1.78)	0.82	0.99 (0.93–1.07)

Abbreviations: BMI, Body Mass Index; CAVAS, Cardiovascular Disease Association Study; HEXA, Health Examinee; KMCC, Korean Multi-center Cancer Cohort; KoGES, Korean Genome Epidemiology Study. [a]Model 1: Stratified by age (20 to <50, 50 to <60, 60 to <70, and 70+ years) and adjusted for age at baseline (continuous, years). [b]Model 2: Model 1 further adjusted for survey year (continuous, year), BMI (10 to <23, 23 to <25, and 25+ kg/m^2), smoking status (never smoker, past smoker, and current smoker for men; never smoker and ever smoker for women), alcohol drinking frequency (never, 0< to <1 time per week, 1 time per week to <3 times per week, 3 times per week to <1 time per day, and 1+ time per day for men; never, 0< to <1 time per day, and 1+ time per day for women). [c]Model 1: Stratified by age (40 to <50, 50 to <60, and 60+ years) and study cohort (the Ansan and Ansung study, the CAVAS, and the HEXA study) and further adjusted for age at baseline (continuous, year), and total energy intake (continuous, kcal/day) [d]Model 2: Model 1 further adjusted for survey year (continuous, year), BMI (10 to <18.5, 18.5 to <23, 23 to <25, 25 to <30, and 30+ kg/m^2), smoking status (0, 0< to <10, 10 to <20, and 20+ pack-years for men; never smoker, past smoker, and current smoker for women), and alcohol intake (0, 0 to <5, 5 to <15, 15 to <30, and 30+ g/day for men; never drinker, past drinker, and current drinker for women)

Table S7. Relative risks (95% confidence intervals) of gastric cancer risk by sex according to salted fish intake in the Korean cohort studies

Outcome	Salted fish intake			*P* for trend	Per increment of 60 g/day
	Tertile 1	Tertile 2	Tertile 3		
**Men**					
**Incidence**					
**KMCC**					
Median, g/day (range)	0 (0–1.99)	6.98 (4.98–6.98)	30.00 (8.57–120.00)		
Case no.	50	75	68		193
Person-years	15040.28	18789.35	19440.18		53269.81
Model 1[a]	1.00 (reference)	1.27 (0.89–1.82)	1.16 (0.81–1.68)	0.71	1.28 (0.76–2.15)
Model 2[b]	1.00 (reference)	1.12 (0.78–1.63)	1.11 (0.76–1.60)	0.73	1.18 (0.68–2.03)
**Mortality**					
**KMCC**					
Median, g/day (range)	0 (0–1.99)	6.98 (4.98–6.98)	30.00 (8.57–120.00)		
Case no.	13	28	19		60
Person-years	15134.28	18975.53	19665.11		53774.92
Model 1[a]	1.00 (reference)	1.83 (0.94–3.56)	1.35 (0.66–2.74)	0.89	1.68 (0.69–4.11)
Model 2[b]	1.00 (reference)	1.31 (0.66–2.59)	1.13 (0.55–2.34)	>0.99	1.36 (0.51–3.60)
**KoGES**					
Median, g/day (range)	0 (0–0.37)	0.75 (0.40–1.51)	3.17 (1.54–86.48)		
Case no.	49	42	40		131
Person-years	165585.86	168079.07	188513.32		522178.24
Model 1[c]	1.00 (reference)	0.98 (0.65–1.49)	0.75 (0.49–1.16)	0.16	2.36 (0.35–16.09)
Model 2[d]	1.00 (reference)	0.98 (0.64–1.48)	0.69 (0.45–1.07)	0.08	1.53 (0.21–11.38)
**Women**					
**Incidence**					
**KMCC**					
Median, g/day (range)	0 (0–1.99)	6.98 (4.98–6.98)	30.00 (8.57–120.00)		

Case no.	38	38	27		103
Person-years	30440.73	34739.92	27189.94		92370.59
Model 1[a]	1.00 (reference)	1.00 (0.63–1.58)	0.95 (0.58–1.56)	0.83	0.74 (0.30–1.83)
Model 2[b]	1.00 (reference)	0.95 (0.59–1.52)	0.91 (0.55–1.49)	0.72	0.67 (0.26–1.70)
**Mortality**					
**KMCC**					
Median, g/day (range)	0 (0–1.99)	6.98 (4.98–6.98)	30.00 (8.57–120.00)		
Case no.	9	14	7		30
Person-years	30556.24	34853.47	27278.51		92688.22
Model 1[a]	1.00 (reference)	1.68 (0.71–3.94)	1.15 (0.42–3.10)	0.97	1.11 (0.24–5.07)
Model 2[b]	1.00 (reference)	1.54 (0.64–3.72)	1.05 (0.38–2.85)	0.84	0.95 (0.19–4.77)
**KoGES**					
Median, g/day (range)	0 (0–0.37)	0.75 (0.40–1.51)	2.89 (1.54–78.11)		
Case no.	23	20	27		70
Person-years	325493.96	296984.55	314362.81		936841.32
Model 1[c]	1.00 (reference)	1.11 (0.60–2.03)	1.25 (0.70–2.23)	0.47	1.14 (0.05–25.38)
Model 2[d]	1.00 (reference)	1.10 (0.60–2.02)	1.24 (0.69–2.22)	0.48	0.96 (0.04–22.15)

Abbreviations: BMI, Body Mass Index; CAVAS, Cardiovascular Disease Association Study; HEXA, Health Examinee; KMCC, Korean Multi-center Cancer Cohort; KoGES, Korean Genome Epidemiology Study. [a]Model 1: Stratified by age (20 to <50, 50 to <60, 60 to <70, and 70+ years) and adjusted for age at baseline (continuous, years). [b]Model 2: Model 1 further adjusted for survey year (continuous, year), BMI (10 to <23, 23 to <25, and 25+ kg/m^2), smoking status (never smoker, past smoker, and current smoker for men; never smoker and ever smoker for women), alcohol drinking status (never, 0< to <1 time per week, 1 time per week to <3 times per week, 3 times per week to <1 time per day, and 1+ time per day for men; never, 0< to <1 time per day, and 1+ time per day for women). [c]Model 1: Stratified by age (40 to <50, 50 to <60, and 60+ years) and study cohort (the Ansan and Ansung study, the CAVAS, and the HEXA study) and further adjusted for age at baseline (continuous, year), and total energy intake (continuous, kcal/day). [d]Model 2: Model 1 further adjusted for survey year (continuous, year), BMI (10 to <18.5, 18.5 to <23, 23 to <25, 25 to <30, and 30+ kg/m^2), smoking status (0, 0< to <10, 10 to <20, and 20+ pack-years for men; never smoker, past smoker, and current smoker for women), and alcohol intake (0, 0 to <5, 5 to <15, 15 to <30, and 30+ g/day for men; never drinker, past drinker, and current drinker for women)

Table S8. Sensitivity analysis of gastric cancer risk according to pickled vegetable intake in the Korean cohort studies

Outcome	Pickled vegetable intake					*P* for trend	Per increment of 40 g/day
	Quintile 1	Quintile 2	Quintile 3	Quintile 4	Quintile 5		
**Incidence**							
**KMCC**							
Case no.	15	15	18	21	12		81
Person-years	9153.04	8983.91	9774.26	11020.96	7474.97		46407.13
RR (95% CI)[a]	1.00 (reference)	1.00 (0.49–2.05)	1.22 (0.61–2.43)	1.12 (0.57–2.19)	0.97 (0.45–2.09)	0.92	0.96 (0.81–1.14)
**Mortality**							
**KoGES**							
Case no.	35	34	31	49	50		199
Person-years	280471.68	282869.54	283603.52	289748.48	314506.54		1451199.76
RR (95% CI)[b]	1.00 (reference)	0.83 (0.52–1.34)	0.79 (0.49–1.29)	1.14 (0.73–1.79)	0.85 (0.54–1.35)	0.85	0.99 (0.95–1.03)

Abbreviations: BMI, Body Mass Index; RR, Relative Risk; CI, Confidence Interval; CAVAS, Cardiovascular Disease Association Study; HEXA, Health Examinee; KMCC, Korean Multi-center Cancer Cohort; KoGES, Korean Genome Epidemiology Study. [a]Stratified by age (20 to < 50, 50 to < 60, 60 to < 70, and 70+ years) and adjusted for age at baseline (continuous, years), sex, survey year (continuous, year), BMI (10 to < 18.5, 18.5 to < 23, 23 to < 25, 25 to < 30, and 30+ kg/m^2), smoking status (never smoker, past smoker, and current smoker), alcohol drinking frequency (never, 0 < to < 1 time per week , 1 time per week to <3 times per week, 3 times per week to < 1 time per day, and 1+ time per day), and fresh vegetable intake (< 1 time per month, 1 time per month to < 1 time per day, and 1+ time per day). [b]Stratified by age (40 to < 50, 50 to < 60, and 60+ years) and study cohort (the Ansan and Ansung study, the CAVAS, and the HEXA study) and further adjusted for age at baseline (continuous, year), sex, total energy intake (continuous, kcal/day), survey year (continuous, year), BMI (10 to < 18.5, 18.5 to < 23, 23 to < 25, 25 to < 30, and 30+ kg/m^2), smoking status (0, 0 < to < 10, 10 to < 20, and 20+ pack-years), alcohol intake (0, 0 < to < 5, 5 to < 15, 15 to < 30, and 30+ g/day), and fresh vegetable intake (g/day in tertile).

Table 9. Characteristics of studies excluded from the meta-analysis.

First author, year	Study	Country	Recruitment period/ follow-up period	Exposure assess-ment	Exposure	Outcome	Cases/total participants	Adjusted variables	Reason for exclusion
Guo W et al., 1994 [1]	The Linxian Nutrition Intervention Trial (NIT Cohort)	China	1985/ Until 1991	FFQ	Pickled vegetables	Incidence	539/29,584	Years of smoking and cancer history in first degree relatives	Superseded by Tran GD et al. [2]
Botterweck AAM et al., 1998 [3]	The Netherlands Cohort Study (NLCS)	Netherla nds	1986–1992/6.3 years	150-item semi-quantitati ve FFQ	Ghrekins	Incidence	265/120,852	Age, sex, smoking, education, stomach disorders, family history of stomach cancer, total fruit consumption, and total vegetable consumption	Exposure specified to particular pickled vegetable
Kobayashi M et al., 2002 [4]	The Japan Public Health Center-based prospective Study (JPHC I)	Japan	1990/ Until 1999	44-item FFQ	Pickled vegetables	Incidence	404/39,993	Age, gender, area, educational level, smoking status, BMI, alcohol intake, use of vitamin A, C, E supplement, total energy intake, highly salted food intake, history of peptic ulcer and family history of gastric cancer	Superseded by Takachi R et al. [5]
Fujino Y et al., 2002 [6]	The Japan Collaborative Cohort Study (JACC)	Japan	1988–1990/ Until 1997	FFQ	Pickles	Mortality	379/44,930	Age	Superseded by Iso H et al. [7]
Tokui N et al., 2005 [8]	The Japan Collaborative	Japan	1988–1990/ Until 1999	33-item FFQ	Pickles, dried or salty fish	Mortality	859/110,792	Age	Superseded by Iso H et al. [7]

	Cohort Study (JACC)								
Kurosawa M et al., 2006 [9]	Higashi-Yamanashi Cohort Study	Japan	1989/ Until 1999	29-item FFQ	Pickled vegetable	Mortality	76/8,035	Age and sex	Superseded by Iso H et al. [7]
Sjodahl K et al., 2008 [10]	Nord-Trondelag Health Study (HUNT I)	Norway	1984–1986/ Until 2002	Question-naire	Salted food (Salted meat or salted fish/ herring)	Incidence	131/73,133	Age, smoking, alcohol drinking, physical activity, and occupation	Exposure includes both salted fish and salted meat
Ko KP et al., 2013 [11]	Korean Multi-Center Cancer Cohort (KMCC)	Korea	1993–2004/ Until 2008	Question-naire	Salted fish	Incidence	166/9,724	Age, sex (in combined analysis), cigarette smoking, BMI, alcohol drinking, and area of residence	Superseded by the present analysis

Abbreviation: FFQ, Food Frequency Questionnaire.

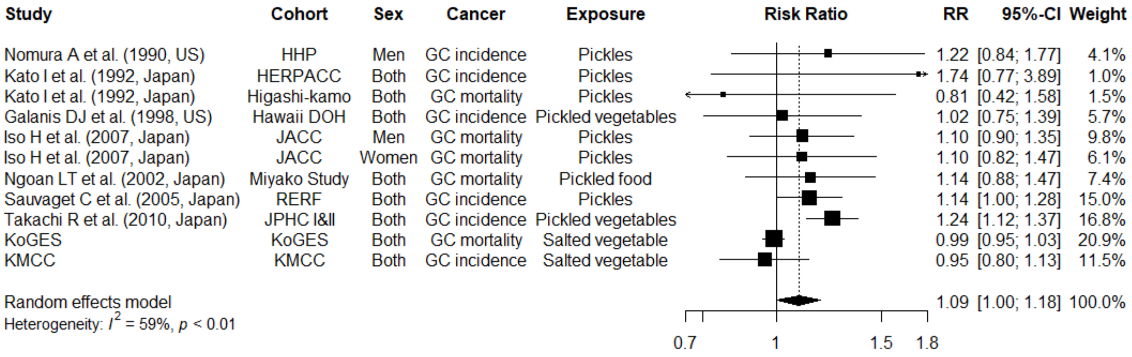

(**a**)

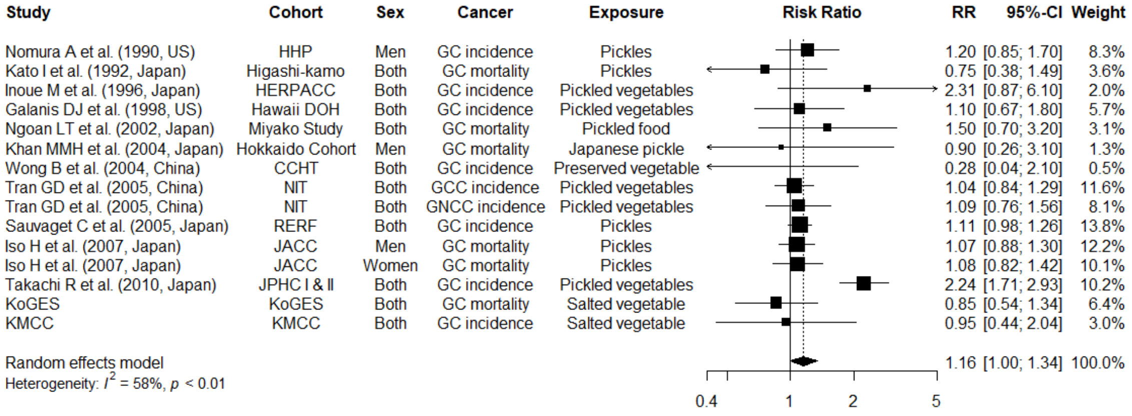

(**b**)

Figure S1. Study-specific and combined relative risks (95% confidence intervals) of overall gastric cancer risk (incidence and mortality combined) (**a**) per 40 g/day increment in pickled vegetable intake and (**b**) comparing the highest to the lowest intake of pickled vegetables; Abbreviation: GC, Gastric Cancer; GCC, Gastric Cardia Cancer; GNCC, Gastric Non Cardia Cancer; RR, Relative Risk; CI, Confidence Interval; CCHT, Changle County Helicobacter Trial; Hawaii DOH, Hawaii Department of Health Survey; HHP, Honolulu Heart Program; HERPACC, Hospital-based Epidemiologic Research Program at Aichi Cancer Center; JACC, Japan Collaborative Cohort Study for Evaluation of Cancer; JPHC, The Japan Public Health Center-based prospective Study; KMCC, Korean Multi-center Cancer Cohort; KoGES, Korean Genome and Epidemiology Study; NIT, Linxian General Population Trial Cohort; RERF, Radiation Effects Research Foundation.

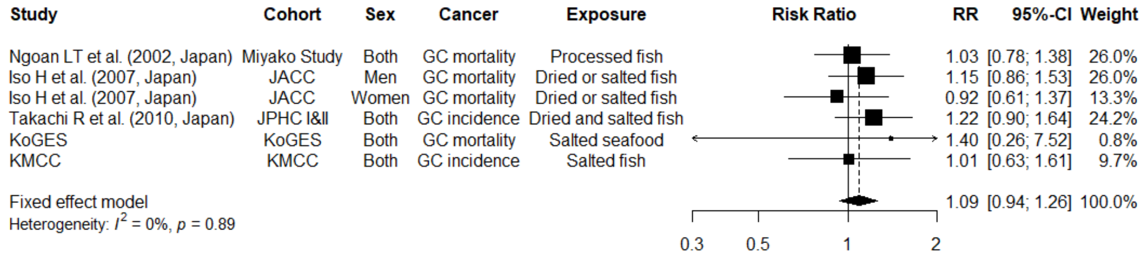

(**a**)

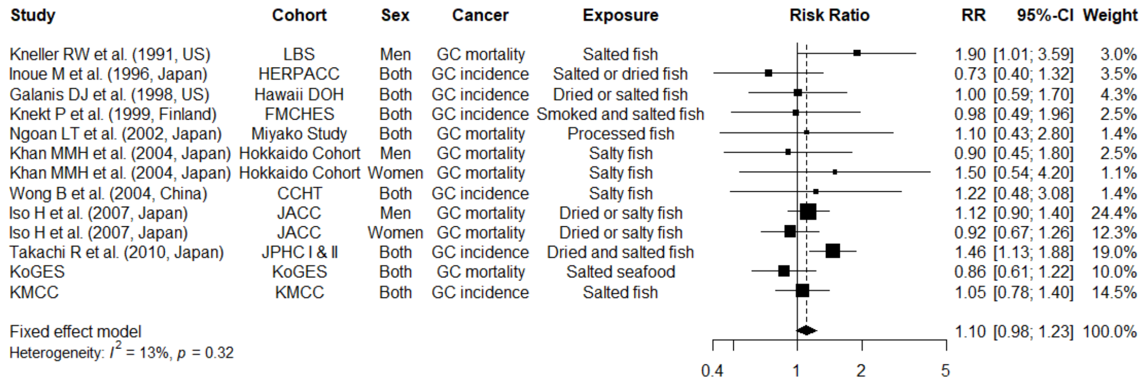

(**b**)

Figure S2. Study-specific and combined relative risks (95% confidence intervals) of overall gastric cancer risk (incidence and mortality combined) (**a**) per 60 g/day increment in salted fish intake and (**b**) comparing the highest to the lowest intake of salted fish; Abbreviation: GC, Gastric Cancer; RR, Relative Risk; CI, Confidence Interval; CCHT, Changle County Helicobacter Trial; FMCHES, Finnish Mobile Clinic Health Examination Survey; Hawaii DOH, Hawaii Department of Health Survey; HERPACC, Hospital-based Epidemiologic Research Program at Aichi Cancer Center; JACC, Japan Collaborative Cohort Study for Evaluation of Cancer; JPHC, The Japan Public Health Center-based prospective Study; KMCC, Korean Multi-center Cancer Cohort; KoGES, Korean Genome and Epidemiology Study.

Table S10. Stratified analysis of pickled vegetable or salted fish intake with the risk of gastric cancer incidence.

Subgroup	No. of studies	RR (95% CI)		Q test, *p*-value	*P* for difference
		Fixed-effects model	Random-effects model		
**Pickled vegetable intake**					
**Dose-response analysis (per 40 g/day increment)**					
**Sex**					0.71
Men	3	0.98 (0.82, 1.16)	0.98 (0.82, 1.16)	0.42	
Women	2	1.04 (0.81, 1.33)	1.04 (0.81, 1.33)	0.71	
**Publication year**					0.86
Before 2000	3	1.14 (0.91, 1.43)	1.14 (0.91, 1.43)	0.44	
Since 2000	3	1.15 (1.07, 1.24)	1.12 (0.98, 1.28)	0.04	
**Follow-up time**					0.94

<15 years	5	1.16 (1.07, 1.25)	1.12 (0.96, 1.31)	0.08	
≥15 years	1	1.14 (1.00, 1.28)	1.14 (1.00, 1.28)	-	
**Ethnicity**					0.08
Korea	1	0.95 (0.80, 1.13)	0.95 (0.80, 1.13)	-	
Japan	5	1.19 (1.11, 1.28)	1.19 (1.11, 1.28)	0.56	
**High versus low analysis**					
**Sex**					0.78
Men	4	1.13 (0.89, 1.44)	1.13 (0.89, 1.44)	0.95	
Women	3	1.09 (0.69, 1.72)	1.09 (0.69, 1.72)	0.81	
**Publication year**					0.83
Before 2000	3	1.22 (0.92, 1.61)	1.22 (0.92, 1.61)	0.40	
Since 2000	5	1.19 (1.08, 1.31)	1.22 (0.91, 1.63)	<.0001	
**Follow-up time**					0.24
<15 years	6	1.59 (1.32, 1.92)	1.36 (0.90, 2.05)	0.01	
≥15 years	2	1.09 (0.99, 1.21)	1.09 (0.99, 1.21)	0.89	
**Ethnicity**					0.19
Korea, China	3	1.04 (0.87, 1.24)	1.04 (0.87, 1.24)	0.63	
Japan	5	1.25 (1.13, 1.39)	1.42 (1.00, 2.03)	0.0001	
**Salted fish intake**					
**Dose-response analysis (per 60 g/day increment)**					
**Sex**					-
Men	1	1.18 (0.68, 2.03)	1.18 (0.68, 2.03)	-	
Women	1	0.66 (0.26, 1.70)	0.66 (0.26, 1.70)	-	
**Publication year**					-
Before 2000	0	-	-	-	
Since 2000	2	1.15 (0.90, 1.48)	1.15 (0.90, 1.48)	0.50	
**Follow-up time**					-
<15 years	2	1.15 (0.90, 1.48)	1.15 (0.90, 1.48)	0.50	
≥15 years	0	-	-	-	
**Ethnicity**					-
Korea	1	1.01 (0.63, 1.61)	1.01 (0.63, 1.61)	-	
Japan	1	1.22 (0.90, 1.64)	1.22 (0.90, 1.64)	-	
**High versus low analysis**					
**Sex**					0.49
Men	2	1.14 (0.84, 1.54)	1.14 (0.84, 1.54)	0.81	
Women	2	0.90 (0.58, 1.40)	0.90 (0.58, 1.40)	0.96	
**Publication year**					0.21
Before 2000	3	0.90 (0.64, 1.27)	0.90 (0.64, 1.27)	0.70	
Since 2000	3	1.27 (1.05, 1.53)	1.25 (0.98, 1.61)	0.23	
**Follow-up time**					0.77
<15 years	5	1.18 (1.00, 1.40)	1.13 (0.89, 1.43)	0.17	
≥15 years	1	0.98 (0.49, 1.96)	0.98 (0.49, 1.96)	-	
**Ethnicity**					0.78
Korea, China, Europe	3	1.05 (0.81, 1.36)	1.05 (0.81, 1.36)	0.93	
Japan	3	1.26 (1.02, 1.56)	1.10 (0.72, 1.67)	0.07	

Abbreviations: RR, Relative Risk; CI, Confidence Interval.

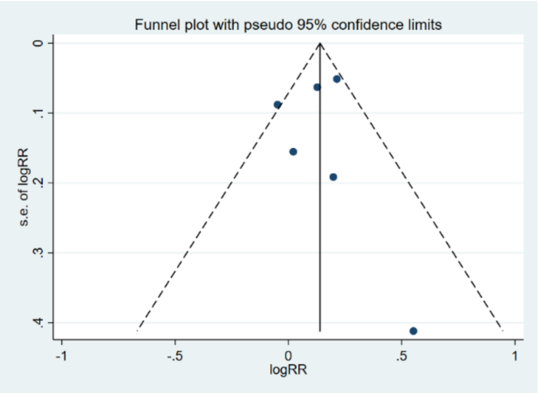

(**a**)

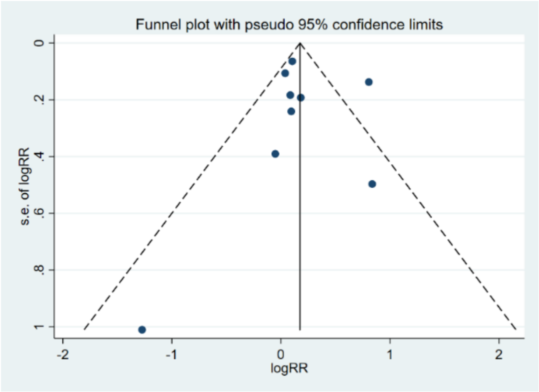

(**b**)

Figure S3. Funnel plot for the studies included in (**a**) dose-response meta-analysis and (**b**) categorical meta-analysis of pickled vegetable intake with gastric cancer incidence.

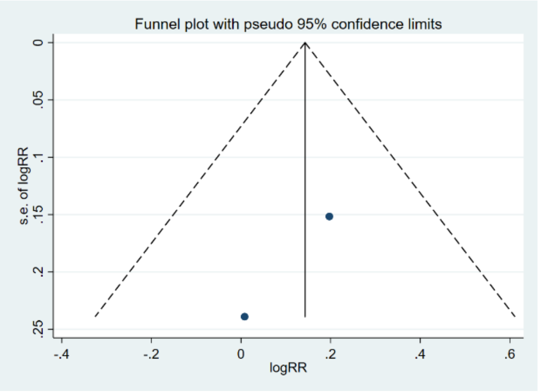

(**a**)

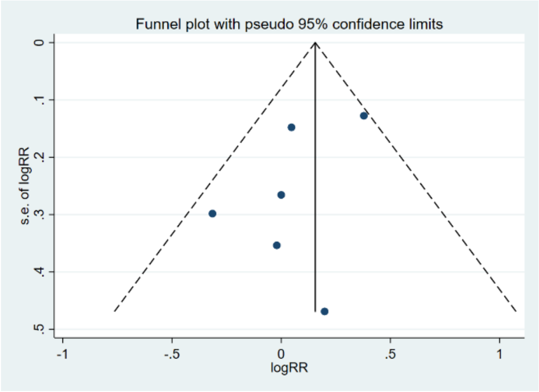

(**b**)

Figure S4. Funnel plot for the studies included in (a) dose-response meta-analysis[a] and (b) categorical meta-analysis of salted fish intake with gastric cancer incidence; [a]Test for publication bias could not be conducted since there were only two observational studies examining the association of gastric cancer incidence with salted fish intake.

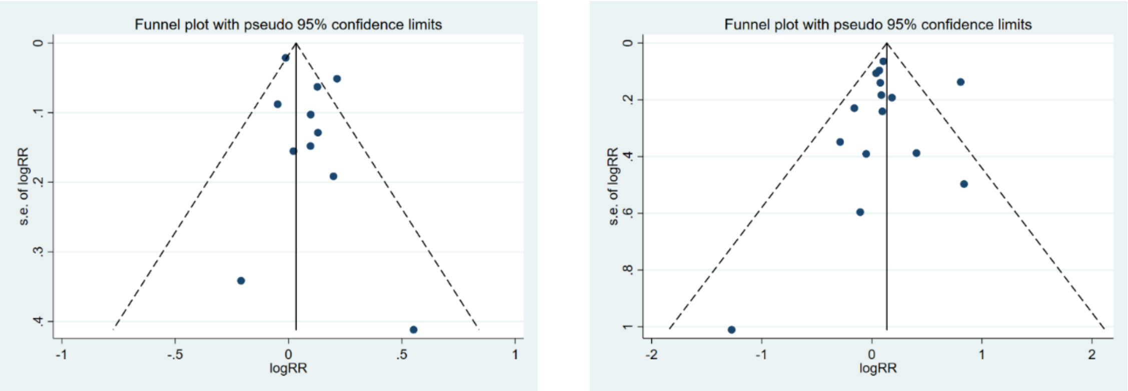

(**a**) (**b**)

Figure S5. Funnel plot for the studies included in (**a**) dose-response meta-analysis and (**b**) categorical meta-analysis of pickled vegetable intake with gastric cancer risk (incidence and mortality combined)**.**

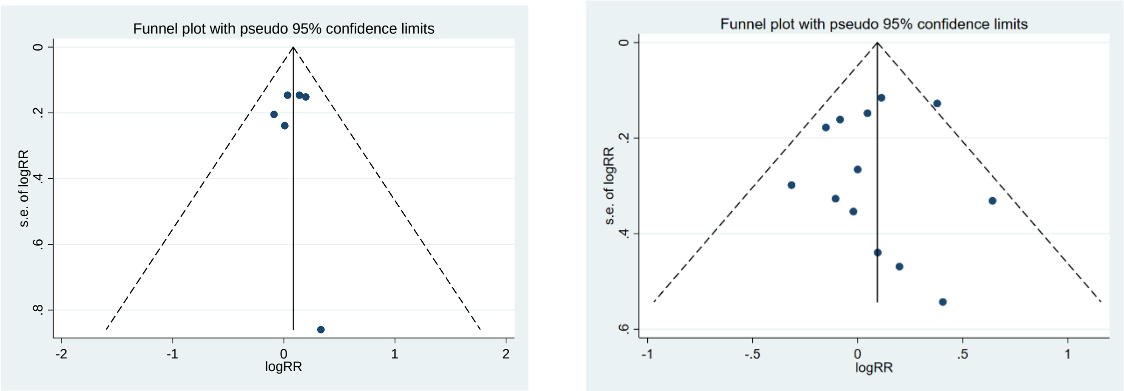

(**a**) (**b**)

Figure S6. Funnel plot for the studies included in (**a**) dose-response meta-analysis and (**b**) categorical meta-analysis of salted fish intake with gastric cancer risk (incidence and mortality combined)

Reference

1. Guo, W.; Blot, W.J.; Li, J.Y.; Taylor, P.R.; Liu, B.Q.; Wang, W.; Wu, Y.P.; Zheng, W.; Dawsey, S.M.; Li, B., et al. A nested case-control study of oesophageal and stomach cancers in the Linxian nutrition intervention trial. *Int. J. Epidemiol.* **1994**, *23*, 444–450, doi:10.1093/ije/23.3.444.
2. Tran, G.D.; Sun, X.D.; Abnet, C.C.; Fan, J.H.; Dawsey, S.M.; Dong, Z.W.; Mark, S.D.; Qiao, Y.L.; Taylor, P.R. Prospective study of risk factors for esophageal and gastric cancers in the Linxian General Population Trial cohort in China. *Int. J. Cancer* **2005**, *113*, 456–463, doi:10.1002/ijc.20616.
3. Botterweck, A.A.; van den Brandt, P.A.; Goldbohm, R.A. A prospective cohort study on vegetable and fruit consumption and stomach cancer risk in The Netherlands. *Am. J. Epidemiol.* **1998**, *148*, 842–853, doi:10.1093/oxfordjournals.aje.a009709.
4. Kobayashi, M.; Tsubono, Y.; Sasazuki, S.; Sasaki, S.; Tsugane, S. Vegetables, fruit and risk of gastric cancer in Japan: a 10-year follow-up of the JPHC Study Cohort I. *Int. J. Cancer* **2002**, *102*, 39–44, doi:10.1002/ijc.10659.
5. Takachi, R.; Inoue, M.; Shimazu, T.; Sasazuki, S.; Ishihara, J.; Sawada, N.; Yamaji, T.; Iwasaki, M.; Iso, H.; Tsubono, Y., et al. Consumption of sodium and salted foods in relation to cancer and cardiovascular disease: the Japan Public Health Center-based Prospective Study. *Am. J. Clin. Nutr.* **2010**, *91*, 456–464, doi:10.3945/ajcn.2009.28587.

6. Fujino, Y.; Tamakoshi, A.; Ohno, Y.; Mizoue, T.; Tokui, N.; Yoshimura, T. Prospective study of educational background and stomach cancer in Japan. *Prev. Med.* **2002**, *35*, 121–127, doi:10.1006/pmed.2002.1066.
7. Iso, H.; Kubota, Y. Nutrition and disease in the Japan Collaborative Cohort Study for Evaluation of Cancer (JACC). *Asian Pac. J. Cancer Prev.* **2007**, *8*, 35–80.
8. Tokui, N.; Yoshimura, T.; Fujino, Y.; Mizoue, T.; Hoshiyama, Y.; Yatsuya, H.; Sakata, K.; Kondo, T.; Kikuchi, S.; Toyoshima, H., et al. Dietary habits and stomach cancer risk in the JACC Study. *J. Epidemiol.* **2005**, *15*, S98–S108, doi:10.2188/jea.15.s98.
9. Kurosawa, M.; Kikuchi, S.; Xu, J.; Inaba, Y. Highly salted food and mountain herbs elevate the risk for stomach cancer death in a rural area of Japan. *J. Gastroenterol. Hepatol.* **2006**, *21*, 1681–1686, doi:10.1111/j.1440-1746.2006.04290.x.
10. Sjodahl, K.; Jia, C.; Vatten, L.; Nilsen, T.; Hveem, K.; Lagergren, J. Salt and gastric adenocarcinoma: a population-based cohort study in Norway. *Cancer Epidemiol. Biomarkers Prev.* **2008**, *17*, 1997–2001, doi:10.1158/1055-9965.epi-08-0238.
11. Ko, K.P.; Park, S.K.; Yang, J.J.; Ma, S.H.; Gwack, J.; Shin, A.; Kim, Y.J.; Kang, D.; Chang, S.H.; Shin, H.R., et al. Intake of soy products and other foods and gastric cancer risk: A prospective study. *J. Epidemiol.* **2013**, *23*, 337–343, doi:10.2188/jea.JE20120232.

© 2020 by the authors. Licensee MDPI, Basel, Switzerland. This article is an open access article distributed under the terms and conditions of the Creative Commons Attribution (CC BY) license (http://creativecommons.org/licenses/by/4.0/).
